# Changes of 5‐hydroxymethyl‐2‐furfural in fresh and processed ginsengs

**DOI:** 10.1002/fsn3.1496

**Published:** 2020-03-18

**Authors:** Yali Li, Yufang Wang, Xiangmin Piao, Peihe Zheng, Hao Zhang, Shifeng Pang, Zhengyi Qu, Yingping Wang

**Affiliations:** ^1^ Institute of Special Wild Economic Animals and Plants Chinese Academy of Agriculture Sciences Changchun Jilin China; ^2^ College of Chinese Medicinal Materials Jilin Agricultural University Changchun Jilin China

**Keywords:** 5‐hydroxymethyl‐2‐furfural, ginseng processing, ultraperformance liquid chromatography–mass spectrometer

## Abstract

The study estimated changes of 5‐hydroxymethyl‐2‐furfuraldehyde (5‐HMF) in different ginseng products with different temperatures and time pretreatment. Heat treatment was performed at various temperatures for 1.50, 2.00, 2.50, and 3.00 hr, respectively. Ultrasonic extraction and reflux extraction were used to evaluate the extraction rate and different solvents (such as 80% methanol, dichloromethane, ethyl acetate, and an extraction with both dichloromethane and ethyl acetate solvents) using two extraction methods (liquid–liquid extraction and solid‐phase extraction) to remove matrix interference. An ultraperformance liquid chromatography–mass spectrometer (UPLC‐MS) method was used for quantitative and changing analysis of 5‐HMF in different ginseng samples. The results indicated that the content of 5‐HMF increased dramatically with heating temperature and time, and the 5‐HMF in the ginseng samples ranged from 0.01 to 112.32 g/kg protein. The highest value was observed in the honey‐added ginseng samples with the highest amount of addition and highest temperature treatment, and the lowest value was found in the fresh ginseng samples. These results implied that 5‐HMF may be as an indicator to estimate the honey addition level and heat treatment degree during the processing of ginseng products, and the content of 5‐HMF is a promising parameter to evaluate the quality of products (ginseng). The production and regulation of potentially harmful Maillard reaction products (PHMRPs)‐5‐HMF in ginseng manufacture will provide an important reference for safe ginseng processing.

## Graphical abstract

The identification of 5‐hydroxymethyl‐2‐furfural (5‐HMF) in ginsengs was confirmed using an ultraperformance liquid chromatography–mass spectrometer (UPLC‐MS) method. The generation and regulation of potentially harmful Maillard reaction products (MRPs) 5‐HMF in ginseng processing are investigated and made suggestions for considering 5‐HMF as an evaluating indicator for quality of ginseng products.

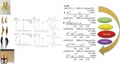

## 1 INTRODUCTION

**Figure 1:**
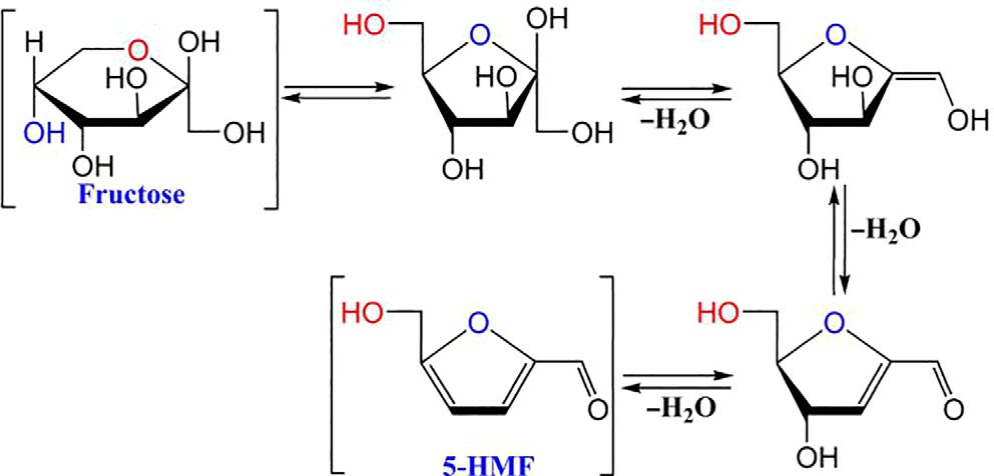
Formation of 5‐HMF from fructose

Hydroxymethyl‐2‐furaldehyde or 5‐hydroxymethyl‐2‐furfuraldehyde (5‐HMF, CAS NO. 67‐47‐0) is one kind of the Maillard reaction products (MRPs) (Hellwig, Lennart Kühn, & Henle, 2018; Imahori et al., 2018; Oliver, Melton, & Stanley, 2006) that occurs in many carbohydrate‐rich foods, traditional Chinese medicines (TCM) and injection, bread, corn syrups (de Andrade et al., (2017), cereal products (Ameur, Trystram, & Birlouez‐Aragon, 2006), biscuits (Švecová & Mach, 2017), honey (Kowalski & Lukasiewicz, 2017), Schisandra chinensis Fructus, Rehmannia glutinosa, DangShen, and Shengqifuzheng injection (Kowalski, Lukasiewicz, Duda‐Chodak, & Zięć, 2003). It is mainly produced by acid catalysis and thermal dehydration of hexoses via 1,2‐enolisation, appearing in products where water coexists with saccharides (Puignou, 2006). The scheme for generation of 5‐HMF from the fructose is shown in Figure 1. In the past decades, there have been a few debates concerning the 5‐HMF’s biological activity. It has been reported that 5‐HMF has biological effects such as antioxidant activity (Zhao et al., 2013), antihypoxia (Li et al., 2011), and inhibition of sickling of red blood cells (Janzowski, Glaab, Samimi, Schlatter, & Eisenbrand, 2000). However, the formation of 5‐HMF could reduce the content of sugar and lessen the nutrient composition. However, numerous studies have raised toxicological concern on 5‐HMF, and its derivatives, 5‐sulfooxymethylfurfural and 5‐chloromethylfurfural, have been found to be genotoxic, cytotoxic, carcinogenic, and mutagenic, inducing skin and hepatic cancers (Durling, Busk, & Hellman, 2009; Ito et al., 2013; Pereira, Albuquerque, Ferreira, Cacho, & Marques, 2011). Furthermore, recent reports have shown that 5‐HMF is a mutagen promoter, which is an important biomarker of carcinogenicity and genotoxicity in mouse cell lines (Glatt, Schneider, & Liu, 2005; Janzowski et al., 2000).

5‐HMF is an important parameter conferring the quality and freshness of some foods, TCM, and injections; therefore, the analysis and control of 5‐HMF have been used to estimate the processing strategies, quality, and organoleptic characteristics of the products. For instance, the existence of 5‐HMF has been used in different foodstuffs, as a good indicating parameter to estimate the storage time or temperature (Rada‐Mendoza, Sanz, Olano, & Villamiel, 2004). The concentration of 5‐HMF in juice and honey has been limited strictly (Arribas‐Lorenzo & Morales, 2010; Gidamis, Chove, Shayo, Nnko, & Bangu, 2004; Monakhova & Lachenmeier, 2012). For instance, the Codex Alimentarius of the World Health Organization and the European Union Directive 110/2001 have established a maximum 5‐HMF quality standard of 40 mg/kg in honey and 50 mg/kg in apple juice as heat treatment and deterioration indicator (Gaspar & Lopes, 2009).

Ginseng has been used as a precious herbal medicine and dietary supplement for thousands of years in China, Western countries, and Korea (Paik & Lee, 2015; Wan et al., 2015). Processing of ginseng has great influence on the active ingredients and pharmacological effects, so the processing of ginseng is very important for ginseng's usual consumption and medical functions (Lee, Lee, Kim, Hong, & Kim, 2015). Processing ginseng not only produced numerous functional substances, but also generated a small amount of potentially harmful ingredients which cannot be ignored, especially the HPMRPs such as 5‐HMF (Favreaufarhadi, Pecukonis, & Barrett, 2015; Wong, Cheng, & Wang, 2012). Due to the abundant amino and carbonyl compounds (such as ginsenosides, amino acids, proteins, or reducing sugars) in the ginseng, the production of 5‐HMF tends to rise while processing (steaming, drying parameters, and honey addition), and the content of 5‐HMF is a useful indicator/parameter to evaluate the browning reaction extension. 5‐HMF is considered to be an important parameter for the quality of different foods and medicines. However, the presence and changes of 5‐HMF in different ginseng products and suggestions to consider 5‐HMF as a promising indicator for quality of ginseng products have not been reported.

Several methodologies such as classical spectrophotometeric techniques (Jr, 1979), liquid chromatography with ultraviolet detection (UV), and photodiode array (DAD) (de Andrade et al., 2016; Ciulu et al., 2013), capillary zone electrophoresis–tandem ion trap mass spectrometry method (CZE‐MS) (Bignardi, Cavazza, & Corradini, 2014), gas chromatography–mass spectrometry (GC–MS), and liquid chromatography–mass spectrometry (LC–MS) have been developed currently for the analysis of 5‐HMF (Puignou, 2006; Gaspar & Lopes, 2009; Teixidó, Moyano, Santos, & Galceran, 2008). LC–MS is a good tool to ensure the unequivocal identification and quantification of 5‐HMF in pending text samples.

In this study, changes of 5‐HMF in different ginseng samples with different temperatures and time pretreatment were investigated. Heat treatment was performed at different temperatures for 1.50, 2.00, 2.50, and 3.00 hr, respectively; subsequently, reflux extraction and ultrasonic extraction were used to evaluate the extraction rate, and different solvents (such as 80% methanol, dichloromethane, ethyl acetate, and a extraction with both the above solvents) using two extraction methods (liquid–liquid extraction and solid‐phase extraction) were used to remove matrix interference. Consequently, a reliable ultraperformance liquid chromatography–mass spectrometer (UPLC‐MS) method was proposed for qualitative and quantitative analysis of the 5‐HMF in various ginseng samples. At the same time, the production and regulation of 5‐HMF in ginseng processing are also analyzed. To our knowledge, few references have been reported in the literature involving the investigation of 5‐HMF changes in ginseng products. Quality parameters were established, and the proposed methodology was applied to investigate the changes of 5‐HMF in fresh and processed ginseng samples, which will provide an important reference for safe ginseng processing.

## 2 EXPERIMENTALS

### 2.1 Materials and methods

Ginseng samples with different growth number of years (3, 4, 5, and 6) were purchased from local markets in Ji'an, China. 5‐HMF standard was purchased from Sigma Company. Solid‐phase extraction (SPE) columns (Oasis HLB 3 cm^3^/60 mg) were purchased from Waters. Nylon purification kits with pore size of 0.45‐μm cutoff were purchased from Massachusetts, USA. Methanol and acetonitrile with HPLC grade were purchased from Fisher Scientific. Milli‐Q (Millipore) water was used in all experiments. Extract solvents such as methanol, ethyl acetate, dichloromethane, and other chemicals were of HPLC or analytical grade. Different heating temperatures (such as 90°C, 110°C, 120°C, and 130°C) and different heating time (such as 1.50, 2.00, 2.50, and 3.00 hr) were performed, respectively, to prepare different ginseng samples.

### 2.2 Sample preparation

Each ginseng sample about 5.0 g was prepared for the next 5‐HMF detection. Add about 50 ml 80% methanol to reflux extract and ultrasonic extract individually. Different solvents (such as dichloromethane, ethyl acetate, and extraction with both) using two extraction methods (liquid–liquid extraction and solid‐phase extraction) were used to remove the interference. The UPLC‐MS method was explored to study the changes of 5‐HMF for each ginseng samples.

### 2.3 Protein‐level analysis

Protein level in ginseng samples was measured on a Dumas Nitrogen Analyzer (Velp NDA 701‐Monza) by the previously reported method (Li, Liu, Men, & Wang, 2018).

### 2.4 Qualitative analysis of 5‐HMF

To identify the presence of 5‐HMF in different ginseng samples, UPLC‐MS analysis was carried on a UPLC/XEVO TQ with electrospray ionization (EI) source (Waters), and a 2 ml aliquot of the prepared sample solution was used for the UPLC‐MS analysis. The separation was achieved on a T_3_ column (2.1 × 100 mm, 1.7 mm, Waters) with a mobile phase consisting of acetonitrile solution as solvent A and water as solvent B. An equivalent elution system was 10% A and 90% B at a total flow rate of 0.3 ml/min. The column temperature was kept constant at 35°C.

### 2.5 Changes of 5‐HMF in ginsengs

The sample extractions were subjected to quantitative analysis, and the quantitative analysis was performed in an external standard method. The UPLC‐MS worked in an EI mode under atmospheric pressure and positive polarity (API‐ES). Other UPLC‐MS conditions were as same as the qualitative analysis of 5‐HMF. The ion mode at selective monitoring was set as *m*/*z* 127.0317, corresponding to 5‐HMF “[M + H]^+^.” Every sample run for 3.5 min, and it returned to the initial conditions by setting a balance time of about 1.0 min before each measurement for balancing the system, which ensures a good repeatability of the methodologies. Results were used as g/kg protein, and all the measurements were operated in triplicates.

## 3 RESULTS AND DISCUSSION

### 3.1 Optimization of the extraction and clean‐up procedure

Several experiments were carried out to develop an optimized extraction and clean‐up strategy to gain clean ginseng extracts prior to UPLC‐MS measurement. 80% methanol was used for reflux extraction and ultrasonic extraction; subsequently, dichloromethane, ethyl acetate, and an extraction with both solvents were used to select the suitable clean‐up procedure. In all conditions, 80% methanol reflux extraction was for 3 hr, and the best extraction yield was received when using dichloromethane by liquid–liquid extraction.

### 3.2 Quantitative analysis of 5‐HMF

#### 3.2.1 Identification of 5‐HMF by UPLC‐MS

**Figure 2:**
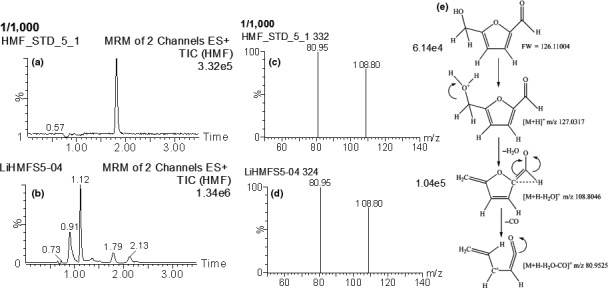
Selective ion monitoring of 5‐HMF by UPLC‐MS. Standard (a); red ginseng at 100°C (b); MS/MS (+) ESI spectra of fragmentation pattern in a solution of 5‐HMF standard (c); and red ginseng at 100°C (d); and the potential MS/MS fragmentation of the compound (e)

The contrast of the mass fragments and retention times for the extraction solution of the ginseng products with those of the reference 5‐HMF indicated the existence of 5‐HMF in the ginseng samples. MS detection used positive multiple reactions monitoring (MRM) mode. The detection conditions were optimized as follows: source temperature, 56°C; drying gas flow, 1,000 L/hr; drying gas temperature, 450°C; collision gas flow, 0.16 ml/min; and nebulizer gas flow, 50 L/hr; Figure 2 shows the UPLC‐MS chromatographs of the reference 5‐HMF and a typical red ginseng sample (a the standard and b red ginseng). The selective ion monitoring fraction of mass spectral analysis for the 5‐HMF standard and the ginseng samples had the same parent/daughter ion patterns of 127.0317, 80.9525, and 108.8046 (Figure 2c and d), and the assignment of the potential MS/MS fragmentation of the constituent was shown in Figure 2e; these results were in good consistency with the previous report for wines (Serra‐Cayuela et al., 2013), certifying the existence of the 5‐HMF in the ginseng samples.

#### 3.2.2 Confirmation of 5‐HMF by ultraviolet scanning

**Figure 3:**
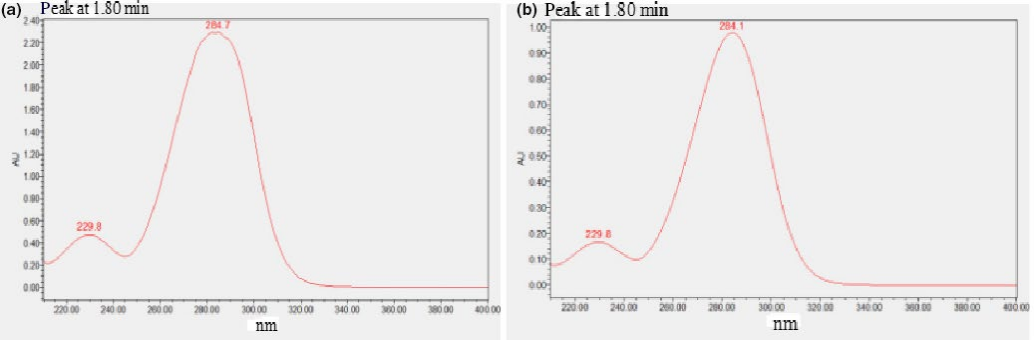
UV spectrum of the compound corresponding to the peak at 1.80 min on UPLC fingerprint of the 5‐HMF standard (a) and red ginseng at 100°C (b)

The red ginseng has the same UV absorption as the commercial 5‐HMF standard at about 1.8 min in the UV spectrum (Figure 3a and b), and the other ginseng samples have the same absorption. The results further confirmed that the 5‐HMF was existed in the different ginseng samples.

### 3.3 Quantitative analysis of 5‐HMF

#### 3.3.1 UPLC‐MS analysis of 5‐HMF

A series of 5‐HMF standard solutions (0.05–5.0 mg/ml) were filtered and then analyzed by the UPLC‐MS system. A good linearity was achieved at concentration range from 0.10 to 3.5 mg/ml with an equation of *Y* = 1.45561 × 106*X* + 100.91407 (*R* ^2^ = 0.99999). The detection limit (DL) of 5‐HMF (three signal‐to‐noise ratio, S/N = 3) was 0.03 mg/ml, and the quantitation limit (QL) was 0.10 mg/ml.

#### 3.3.2 Precision and recoveries

The 5‐HMF concentrations in fresh ginseng and red ginseng samples were observed by the peak area calculation strategy, and RSD values of the 5‐HMF concentrations were 2.28%, 2.30%, 1.97%, 1.83%, 2.65%, and 3.06% by six parallel measurements. The results showing the established method had good precision for 5‐HMF analysis. To evaluate accuracy of the method, the recovery of 5‐HMF was studied by spiking a mixture of standard 5‐HMF (1 to 3 times of the sample's concentrate) into the red ginseng sample. As shown in Table 1, the standard deviations for each spiked sample were less than 3%, thus approving the absence of matrix effects and the accuracy of the detection.

**Table 1:** Standard deviations for three replicates of each spiked sample of 5‐HMF in red ginseng

Samples	Original (mg/ml)	3 replicates	Spiked (mg/ml)	Found (mg/ml)	Standard deviation $\bar{R}$ (%)
Red ginseng	0.10	A1	0.10	0.20	2.08
		A2	0.10	0.23	
		A3	0.10	0.19	
		B1	0.15	0.26	3.00
		B2	0.15	0.23	
		B3	0.15	0.28	
		C1	0.20	0.30	2.52
		C2	0.20	0.33	
		C3	0.20	0.28	
		D1	0.25	0.38	2.08
		D2	0.25	0.37	
		D3	0.25	0.34	
		E1	0.30	0.43	2.65
		E2	0.30	0.38	
		E3	0.30	0.42	

#### 3.3.3 Changes of 5‐HMF in ginsengs

**Figure 4:**
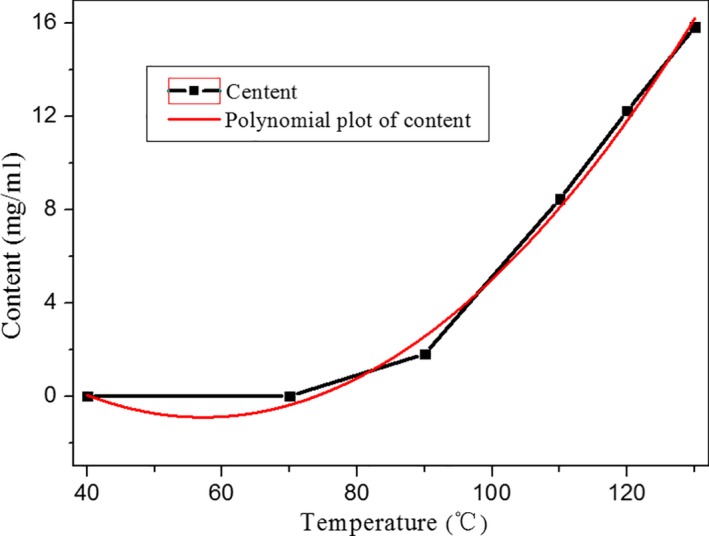
Contents of the 5‐HMF for heated ginseng with 5 years of age at various temperatures

**Figure 5:**
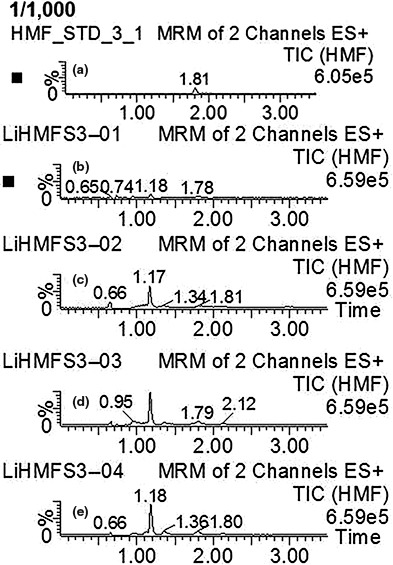
Chromatograms of standard HMF (a), 3 years (b), 4 years (c), 5 years (d), and 6 years (e) ginseng heated at 100°C for 2.5 hr

Quantitative detection results of 5‐HMF in heated 5‐year‐old ginseng at different temperatures for 2.5 hr under the given UPLC‐MS conditions are shown in Figure 4 and Table 2. It was found that the content of 5‐HMF in processed ginsengs increased significantly when the temperature exceeds about 100°C. It indicates that temperature should be set lower than 100°C when we fabricate the red ginseng products. Chromatograms of reference 5‐HMF and ginseng samples with different growth years heated at 100°C for 2.5 hr indicated that the content of 5‐HMF was increasing with the age of ginseng (Figure 5). Monitoring the content of 5‐HMF during the processed red ginseng indicated that the generation of 5‐HMF was slow during the steaming treatment, obtaining values of 3.09 to 12.24 g/kg protein (Table 3).

**Table 2:** 5‐HMF value of heated 5‐year‐old ginseng at various temperatures

Samples	Treatment	HMF content (mg/kg)
5‐year‐old ginseng	40°C, 2.5 hr	0.015
5‐year‐old ginseng	70°C, 2.5 hr	0.02
5‐year‐old ginseng	90°C, 2.5 hr	1.82
5‐year‐old ginseng	110°C, 2.5 hr	8.46
5‐year‐old ginseng	120°C, 2.5 hr	12.25
5‐year‐old ginseng	130°C, 2.5 hr	15.85

**Table 3:** Contents of 5‐HMF in red ginsengs of 5 years when heating at 100°C for 1.0, 1.5, 2.0, 2.5, and 3.0 hr

Samples	Treatment	HMF content (mg/kg)
5‐year‐old ginseng	100°C, 1.0 hr	3.09
5‐year‐old ginseng	100°C, 1.5 hr	5.34
5‐year‐old ginseng	100°C, 2.0 hr	6.80
5‐year‐old ginseng	100°C, 2.5 hr	8.29
5‐year‐old ginseng	100°C, 3.0 hr	12.24

Table 4 shows the values of 5‐HMF content in red ginsengs of 5 years old which were dried at different temperatures after steaming at 100°C for 2.5 hr. The value of 5‐HMF varied from 13.29 to 28.16 g/kg protein. The content of 5‐HMF increased significantly when the drying temperature was higher than 70°C. The 5‐HMF content increased during the heating process, reaching the values of 12.15, 15.99, 22.52, 28.36, and 42.55 g/kg protein after 6, 12, 18, 24, and 30 hr at 70°C, respectively (Table 5). The formation of 5‐HMF was mainly in the air‐heating (drying) process from the data. In addition, the addition of honey to the ginseng increased the 5‐HMF content to 112.32 g/kg protein. Although 5‐HMF was detected in both fresh and processed ginseng samples, its value ranged from 0.01 to 112.32 g/kg protein. These results indicated that ginseng protein was glycosylated such as UHT milk (310–603 g/kg protein) or processed cheese (3.5–366.6 g/kg protein) (Bosch, Alegrı´a, Farré, & Clemente, 2008). The content of 5‐HMF in fresh ginseng is low, but relatively high in honey‐added red ginsengs. The content of 5‐HMF in processed ginseng products depends on many factors such as processing strategies, heating levels, and the excipients, but fresh ginseng is not affected on any of the cases. 5‐HMF is one of the most important PHMRPs from MRs in ginseng, and the processing conditions and the addition of honey to the red ginsengs are more feasible to MRs; thus, the more 5‐HMF was generated. The highest value of 5‐HMF in honey‐added red ginseng shows that honey addition to red ginseng decreased nutritional and medical values, although it improved its organoleptic properties (flavor and taste).

**Table 4:** Contents of 5‐HMF in ginseng of 5 years when steaming at 100°C for 2.5 hr and heating at 40°C, 50°C, 60°C, 70°C, and 80°C, respectively

Samples	Treatment	HMF content (mg/kg)
5‐year‐old ginseng	Steaming at 100°C for 2.5 hr, drying at 40°C for 12 hr	13.29
5‐year‐old ginseng	Steaming at 100°C for 2.5 hr, drying at 50°C for 12 hr	14.35
5‐year‐old ginseng	Steaming at 100°C for 2.5 hr, drying at 60°C for 12 hr	14.52
5‐year‐old ginseng	Steaming at 100°C for 2.5 hr, drying at 70°C for 12 hr	15.99
5‐year‐old ginseng	Steaming at 100°C for 2.5 hr, drying at 80°C for 12 hr	28.16

**Table 5:** Contents of 5‐HMF in ginseng of 5 years when steaming at 100°C for 2.5 hr and heating at 70°C with 6, 12, 18, 24, and 30 hr, respectively

Samples	Treatment	HMF content (mg/kg)
5‐year‐old ginseng	Heating at 70°C, 6 hr	12.15
5‐year‐old ginseng	Heating at 70°C, 12 hr	15.99
5‐year‐old ginseng	Heating at 70°C, 18 hr	22.52
5‐year‐old ginseng	Heating at 70°C, 24 hr	28.36
5‐year‐old ginseng	Heating at 70°C, 30 hr	42.55

## 4 CONCLUSIONS

The study estimated the changes of 5‐HMF in fresh and manufactured ginseng samples. The results indicated that the lowest content of 5‐HMF is found in the fresh ginseng sample, and the highest is found in honey adding red ginsengs with the highest amount of honey addition and temperature treatment. In conclusion, the heat treatment and honey addition dramatically increased the 5‐HMF content in ginsengs. These results implied that the concentration of 5‐HMF may be used as a promising indicator to estimate the honey addition and heating level during ginseng processing. Lower level honey addition and lower temperature should be used to avoid forming the potentially harmful MRPs 5‐HMF during processing of ginseng and other high polysaccharides‐contained foods and herbal medicines. This research provided valuable information for the formation and regulation of 5‐HMF in ginseng processing and also provided a useful reference for safe approach of ginseng processing. In addition, the existence of different values of 5‐HMF in different ginseng samples for this study can serve as a foundation for the standard doses of the ginseng available doses per day.

## CONFLICTS OF INTEREST

The authors have no conflicts of interest.

## AUTHORS’ CONTRIBUTIONS

Yali Li designed the experiments and contributed to the statistical analyses and the writing of the manuscript. Yufang Wang and Xiangmin Piao contributed to the processing of ginsengs. Peihe Zheng and Hao Zhang contributed to the pretreatment of samples. Shifeng Pang and Zhengyi Qu contributed to UPLC‐MS experiments. Yingping Wang put forward the ideas and approved the manuscript. All the authors read and proofed the final manuscript.

## ETHICAL APPROVAL

This study does not involve any human or animal testing.
